# Pathological cell-cell interactions are necessary for striatal pathogenesis in a conditional mouse model of Huntington's disease

**DOI:** 10.1186/1750-1326-2-8

**Published:** 2007-04-30

**Authors:** Xiaofeng Gu, Véronique M André, Carlos Cepeda, Shi-Hua Li, Xiao-Jiang Li, Michael S Levine, X William Yang

**Affiliations:** 1 Center for Neurobehavioral Genetics, Semel Institute for Neuroscience and Human Behavior, David Geffen School of Medicine, University of California at Los Angeles, CA 90095, USA; 2 Mental Retardation Research Center, Semel Institute for Neuroscience and Human Behavior, David Geffen School of Medicine, University of California at Los Angeles, CA 90095, USA; 3 Department of Psychiatry & Biobehavioral Sciences, David Geffen School of Medicine, University of California at Los Angeles, CA 90095, USA; 4 Brain Research Institute, University of California at Los Angeles, CA 90095, USA; 5 Department of Human Genetics, Emory University, Atlanta, GA 30322, USA

## Abstract

A critical issue in understanding Huntington's disease (HD) pathogenesis is how the ubiquitously expressed mutant huntingtin (mhtt) with an expanded polyglutamine repeat can cause selective toxicity of striatal and cortical neurons. Two potential cellular models may contribute to such specificity: expression of mhtt in these vulnerable neurons alone may be sufficient to result in their dysfunction and/or degeneration (cell-autonomous model); or mhtt in other cell types can elicit pathological cell-cell interactions to cause the vulnerable neurons to become dysfunctional and be at risk for degeneration (cell-cell interaction model). To distinguish between these two models, we have selectively expressed a neuropathogenic fragment of mhtt-exon1 in striatal medium spiny neurons (MSNs) by crossing a conditional mouse model of HD with a striatal-specific Cre mouse line. In this striatal model of HD, we observed progressive and cell-autonomous nuclear accumulation of mhtt aggregates in MSNs. Surprisingly, unlike the mouse model expressing mhtt-exon1 in all the neurons in the brain, the striatal model lacks significant locomotor deficits and striatal neuropathology including gliosis and dark degenerating neurons. Electrophysiological findings from acutely dissociated MSNs revealed a cell-autonomous deficit in N-methyl-d-aspartate (NMDA) receptor sensitivity to Mg^2+^, a deficit also present in other mouse models of HD. In conclusion, this study provides the first *in vivo *genetic evidence that pathological cell-cell interactions are necessary for striatal pathogenesis in a conditional mouse model of HD, and suggests a ''two-hit'' hypothesis in which both cell-autonomous toxicity and pathological cell-cell interactions are critical to HD pathogenesis.

## Background

Huntington's disease (HD) is an autosomal dominant neurodegenerative disorder characterized clinically by movement abnormalities (*i.e. *chorea), psychiatric symptoms, and cognitive deficits [1]. HD belongs to a group of neurodegenerative disorders caused by polyglutamine repeat (polyQ) expansion. In HD, the CAG repeat mutation encoding the polyQ repeat lies in exon 1 of the mutant huntingtin gene: the normal repeat is less than 36 while the mutant repeat ranges from 36 to about 120 [2,3]. In all polyglutamine disorders including HD, the polyQ repeat length is inversely correlated with the age of onset of symptoms [4-8].

A critical question in understanding pathogenesis of HD as well as other polyQ disorders is how the widely expressed mutant polyQ protein can cause highly selective neuronal toxicity. In each polyQ disorder, distinct subsets of neurons in the CNS are selectively affected. The most affected neuronal type in HD is the striatal medium spiny neuron (MSN), with the vast majority of MSNs degenerating in the advanced stage of the disease. The second most affected neuronal population in HD is the cortical pyramidal neuron (CPN), particularly those that lie in the deep cortical layers [9,10]. Contrary to the significant neurodegeneration in the striatum and cortex, neurons in the cerebellum and other sub-cortical brain regions are usually spared in the majority of HD patients. The latter brain regions may exhibit some neurodegeneration only in the most severely affected, juvenile-onset HD patients who have relatively longer polyQ repeats [11]. Since mutant huntingtin (mhtt) is ubiquitously expressed in the brain and peripheral tissues [11-13], the selective pattern of neuronal toxicity in HD cannot be explained by the expression pattern of mhtt [14-17].

Conceptually, there are at least two cellular models that could account for the selective neuronal toxicity in HD. In the first, cellular toxicity of mhtt within the vulnerable neurons alone is sufficient to elicit progressive dysfunction and degeneration of these neurons (cell-autonomous model). In the second, pathological cell-cell interactions elicited by mhtt expression in other cell types are necessary or even sufficient to cause the dysfunction and degeneration of the vulnerable neurons (cell-cell interaction model). Full length mhtt as well as its toxic N-terminal fragments, which are known to be generated by proteolysis [9,18-21], can elicit a variety of cell-autonomous toxicities including synaptic dysfunction, defective endocytosis, axonal transport deficits, mitochondria abnormalities, and transcriptional dysregulation [22]. Recent studies also suggest that mhtt can generate pathological cell-cell interactions including aberrant corticostriatal neurotransmission [23], abnormal glial function [24], and reduction of cortical brain derived neurotrophic factor (BDNF) [25] which is neurotrophic to MSNs [26]. Despite the rich literature demonstrating the presence of both cell-autonomous toxicity and pathological cell-cell interactions in HD models, their relative contributions to disease pathogenesis remain poorly defined.

**Figure 1:**
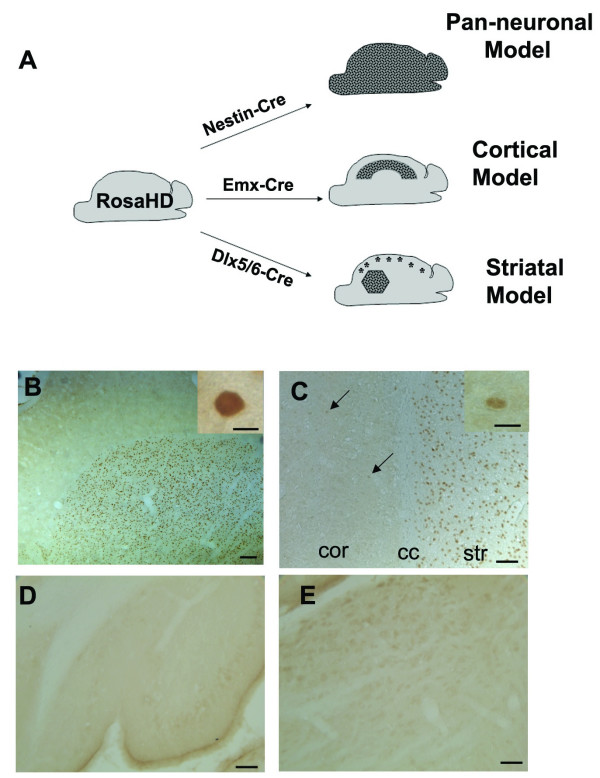
**Generation of the striatal specific mouse model of HD**. (A) Schematics illustrate the strategy to use cell-type-specific Cre to selectively activate mhtt-exon1 expression in all neurons in the brain (pan-neuronal model), only in CPNs (cortical model; [27]), and only in striatal MSNs and a subset of interneurons (asterisks) in the cortex (striatal model). (B) Immunohistochemical staining using polyclonal EM48 antibody, which is specific to aggregated forms of mhtt [9], reveals a cell-autonomous accumulation of nuclear mhtt aggregates in the striatum of the striatal model at 6 months of age. Inset in B shows at a higher magnification the EM48 staining in the nucleus is consisted of diffuse nuclear staining, and not large nuclear inclusions or smaller microaggregates. (C) EM48 staining reveal strong nuclear staining in the striatum (str) but sparse nuclear staining in the cortex (cor), inset in C shows diffuse EM48 nuclear staining of one cell at high magnification. cc: corpus callosum. Finally, there is no nuclear EM48 staining in other brain regions including cerebellum (D) and brain stem (E). Scale bars are 50 μm except in B (200 μm) and insets in B and C (10 μm).

To begin to experimentally address this issue we have developed a Cre/LoxP conditional mouse model of HD (termed RosaHD mice) in which expression of a neuropathogenic fragment of mhtt (mhtt-exon1), driven by the endogenous ubiquitously-expressing Rosa26 promoter, can be switched on by Cre recombinase (Fig. 1A; also ref. [27]). By crossing the RosaHD mouse with the cortex-specific Emx-1 Cre mouse (cortical model) or the pan-neuronal Nestin-Cre mouse (pan-neuronal model), we have obtained direct genetic evidence that cortical pathogenesis in the HD mice requires pathological cell-cell interactions. We found robust locomotor deficits and cortical (and striatal) pathology, including gliosis, dark neuron degeneration and dystrophic neurites, are only observed in the pan-neuronal model but not in the cortical model of HD, despite accumulation of mhtt aggregates in CPNs in both models. Electrophysiological analyses of these models revealed a deficit in cortical inhibition likely due to cortical interneuron dysfunction, suggesting that these interneurons may contribute to cortical pathogenesis in HD [27].

In the current study, we used the RosaHD model to address whether pathological cell-cell interactions are required for striatal pathogenesis *in vivo*. By crossing RosaHD mice with Dlx5/6-Cre mice, we have selectively switched on mhtt-exon1 expression in all striatal MSNs and a subset of cortical interneurons. We found that progressive nuclear accumulation of mhtt aggregates in the striatum is a cell-autonomous process, but the striatal model lacks the locomotor deficit and significant striatal neuropathology which are evident in the pan-neuronal model of HD. However, electrophysiological analyses revealed that both the striatal model and pan-neuronal model exhibit cell-autonomous dysfunctions in NMDA receptor-mediated responses. The present analyses demonstrate that pathological cell-cell interactions are required for striatal pathogenesis in the RosaHD mouse model. Furthermore, our regionally-specific HD mice support a "two-hit" hypothesis in which both cell-autonomous toxicity and abnormal cell-cell interactions contribute to HD pathogenesis *in vivo*.

## Results

### Generation of the striatal model of HD

To selectively express mhtt-exon1 in striatal MSNs, we crossed RosaHD mice with the well-characterized Dlx5/6-Cre mice, which have been shown to mediate LoxP recombination in striatal MSNs, and in a subset of cortical and olfactory interneurons during embryonic development between E11 and P0 [28,29]. To demonstrate the regional specificity of mhtt-exon1 expression in the RosaHD/Dlx5/6-Cre (termed the striatal model), we performed immunostaining in 6 month old double transgenic mice with the EM48 antibody, a rabbit polyclonal antibody raised against the N-terminal of human htt which is highly specific to the aggregated forms of mhtt in HD patients and HD mouse brains [9]. Consistent with our prior observation that mhtt aggregation in the RosaHD cortical and pan-neuronal models is a cell-autonomous process, we detected selective accumulation of EM48 mhtt, in the form of diffuse nuclear staining but not large nuclear inclusions, in the striatum of the striatal model (Fig. 1B). There were a few cells with EM48 diffuse nuclear staining in the cortex (Fig. 1C), but little EM48 nuclear staining was detected in other brain regions including the cerebellum (Fig. 1D) and brain stem (Fig. 1E), *etc*. EM48 (+) staining was not observed in wildtype (WT) littermate controls or in the striatal model in at 1 month of age (data not shown), demonstrating that the diffuse nuclear accumulation of EM48 (+) mhtt in the striatum is a cell-autonomous and progressive process in the striatal model of HD.

**Figure 2:**
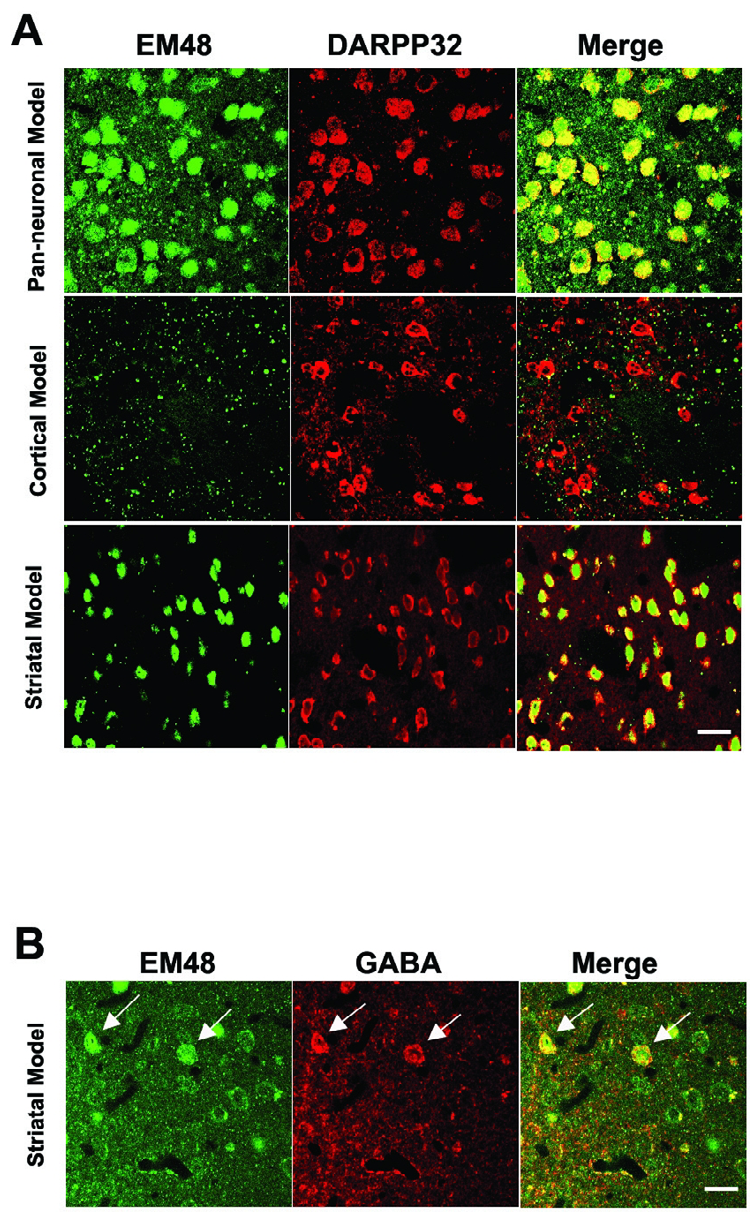
**Selective accumulation of mhtt aggregates in MSNs in the striatal model**. (A) Double immunofluorescent staining with EM48 antibody [30] and anti-Darpp32 (a MSN marker) using 6 month old coronal brain sections from striatal, cortical, and pan-neuronal models of HD. In both striatal and pan-neuronal models the vast majority of MSNs (>98%) have diffuse nuclear EM48 staining. Such a staining pattern is not present in the MSNs in the cortical model or WT mice at this age (data not shown for the WT). (B). Double immunofluorescent staining with EM48 antibody and anti-GABA antibody (marker for cortical interneurons) in the striatal model of HD reveal that a few cells that accumulate nuclear EM48 staining in the cortex are GABA (+) interneurons (white arrows). Scale bars: 100 μm

To further determine the specificity and extent of mhtt-exon1 expression in MSNs in the striatal, cortical and pan-neuronal models of HD, we performed double immunofluorescent staining using EM48 and Darpp-32, a marker for striatal MSNs in 6 month old mice [30]. We observed greater than 96% of the MSNs in the striatal model and 99% of MSNs in the pan-neuronal model also were positive for EM48 (out of more than 200 randomly chosen Darpp-32 positive cells for each genotype), while none of the MSNs in the cortical model or WT controls were positive for EM48 (Fig. 2 and data not shown). Consistent with the prior observation that Dlx5/6-Cre is expressed in the cortical interneuron precursors [27], we found that a subset of cortical GABA positive interneurons also accumulated EM48 (Fig. 2B), but none of the CPNs accumulated EM48 positive nuclear mhtt aggregates in the striatal model (data not shown). In summary, our analyses revealed that the striatal model of HD selectively expresses mhtt in the vast majority of striatal MSNs, and furthermore, the process of mhtt nuclear accumulation and aggregation is also cell-autonomous in this model.

### Lack of significant motor deficits and striatal pathology in the striatal model of HD

**Figure 3:**
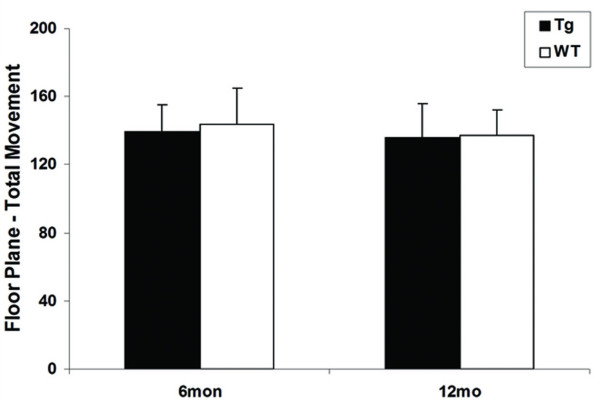
**Lack of progressive locomotor deficits in the striatal model**. In the striatal model, locomotor activity in the open field test is normal at 6 and 12 months, demonstrating cell-autonomous accumulation of mhtt aggregates in MSNs are not sufficient to elicit a motor deficit.

**Figure 4:**
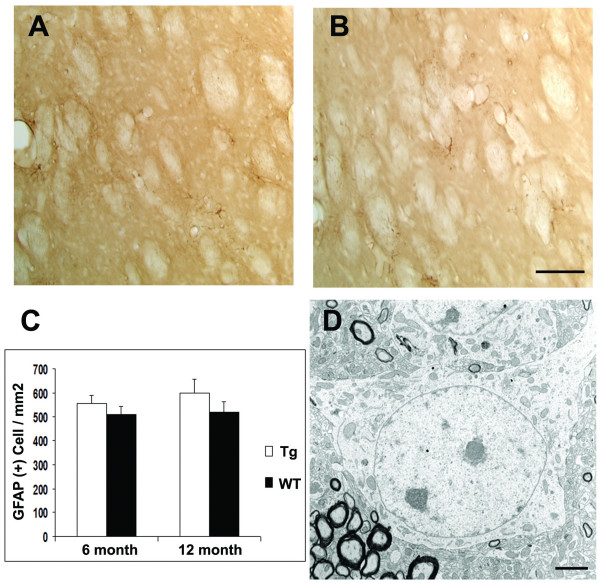
**Lack of robust neuropathology in the striatal model**. In the striatal model, we did not detect gliosis as indicated by the lack of GFAP staining in 12 month old transgenic mice (A) and in WT mice (12 months, B). Quantification of striatal gliosis [27] did not reveal any statistically significant differences between the two genotypes (C). (D) EM analyses did not reveal any degenerating dark neurons in the striatum (N = 2 per genotype). A representative healthy striatal neuron from the striatal model at 12 months of age is shown. Scale bars: A and B 100 μm; D 5 μm.

We next compared the severity of the motor deficits and striatal neuropathology between the pan-neuronal model and the striatal model. Our prior analyses showed that the pan-neuronal model exhibits locomotor hypoactivity at 6 months, striatal gliosis onset at 6 months and dark neuron degeneration at 12 months [27]. In the current study, we used the same battery of tests to evaluate the striatal model. To our surprise, these mice did not exhibit any locomotor deficits at either 6 or 12 months (Fig. 3). Furthermore, we did not detect significant gliosis or dark neurons up to 12 months of age (Fig. 4). Since striatal expression of mhtt-exon1 is comparable in the two models driven by the same endogenous Rosa26 promoter, as demonstrated by EM48 nuclear accumulation in MSNs, our results clearly demonstrate that cell-autonomous toxicity of mhtt in striatal MSNs is not sufficient to elicit robust motor deficits and striatal neuropathology in these mice. These analyses demonstrate, for the first time, that pathological cell-cell interactions are necessary for striatal pathogenesis in a genetic mouse model of HD.

### Cell-autonomous NMDA receptor dysfunction in the striatal model of HD

To further characterize similarities and differences among models we assessed NMDA receptor function in acutely dissociated striatal MSNs in all three models in mice at 6 months of age. First we examined membrane properties of the dissociated neurons. The only significant change in membrane properties occurred in the pan-neuronal model in which membrane capacitance was smaller in the transgenic cells (12.1 ± 0.6 pF in WTs versus 10.8 ± 0.6 pF in transgenics; p < 0.05, t-test), suggesting a smaller neuron size. There was no significant change in capacitance in the cortical and striatal models. Input resistance was similar in WT and transgenic cells in all three groups.

**Figure 5:**
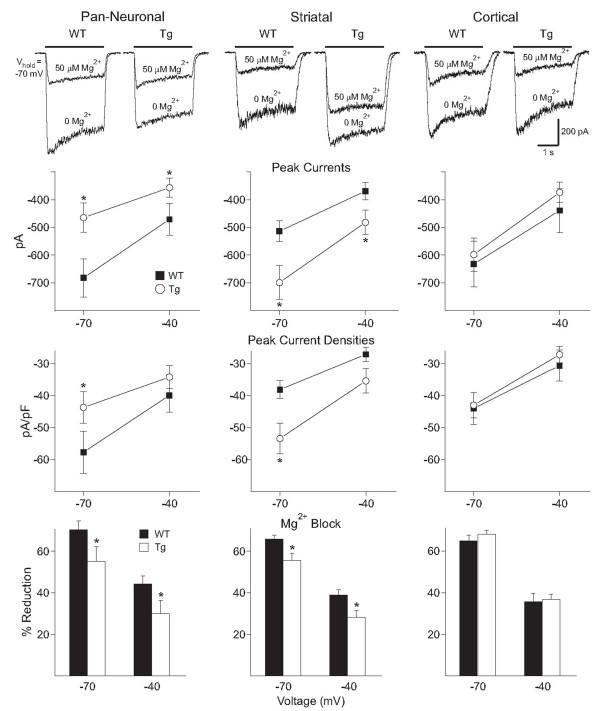
**Alterations in NMDA receptor-mediated currents**. Top panel. Representative traces of currents induced by 100 μM NMDA in the presence or absence of 50 μM Mg^2+ ^at a holding potential of -70 mV. In the pan-neuronal model, the NMDA current in the absence of Mg^2+ ^is smaller in the transgenic (Tg) cell while in the presence of Mg^2+^, the current is similar to the WT. In the striatal model, the NMDA current in the presence or absence of Mg^2+ ^is larger in the transgenic cell than in the WT cell. In the cortical model NMDA currents in the presence or absence of Mg^2+ ^were similar in WT and transgenic cells. 2^nd ^and 3^rd ^Panels. Graphs showing mean peak currents (± SEM) and mean peak current densities (± SEM) evoked by 100 μM NMDA in the absence of Mg^2+ ^in each model. In the pan-neuronal model, NMDA peak currents were significantly smaller in transgenic cells at -70 and -40 mV and current densities were significantly smaller at -70 mV only. In contrast, in the striatal model, NMDA peak currents were significantly larger in transgenic cells at -70 and -40 mV and current densities were significantly larger at -70 mV only. In the cortical model, there were no differences in NMDA peak currents and current densities between WT and transgenic cells at any holding potential. Bottom Panel. Bar graphs showing mean percent block (± SEM) of NMDA currents by 50 μM Mg^2+^. In the pan-neuronal and striatal models, Mg^2+ ^block was significantly smaller in transgenic compared to WT cells at -70 and -40 mV. In the cortical model, there were no differences in Mg^2+ ^block between WT and transgenic cells at any holding potential.

We then determined if there were changes in NMDA receptor-mediated currents. Fig. 5 (top panel) shows typical currents evoked by application of NMDA in neurons from mice in each group. In the absence of Mg^2+^, NMDA current amplitudes and densities were dependent on holding potential and decreased at -40 mV compared to -70 mV in WT and transgenic cells in all 3 groups (ANOVA followed by Bonferroni's t-test p < 0.001, Fig. 5, 2^nd ^and 3^rd ^panel). Co-application of 50 μM Mg^2+ ^with 100 μM NMDA significantly decreased currents in WT and transgenic cells in all groups (ANOVA followed by Bonferroni's t-test p < 0.001, Fig. 5, bottom panel). In the pan-neuronal model, 100 μM NMDA induced significantly smaller peak currents in transgenic compared to WT cells at both -70 and -40 mV holding potentials (ANOVA followed by Bonferroni's t-test p < 0.05) (Fig. 5, 2^nd ^panel). Current densities also were significantly smaller in transgenic cells but only at -70 mV (p < 0.01, Fig. 5, 3^rd ^panel). In the presence of Mg^2+^, NMDA peak currents and densities were no longer different between WT and transgenic cells, at either holding potential (p > 0.05). However, the percent block by Mg^2+ ^was significantly smaller in transgenic compared to WT cells at -70 and -40 mV (p < 0.05, Fig. 5, bottom panel). In contrast to the pan-neuronal model, NMDA peak currents and densities in the striatal model were larger in transgenic compared to WT cells at -70 mV (ANOVA followed by Bonferroni's t-test p < 0.05) (Fig. 5, 2^nd ^and 3^rd ^panels). The difference was not significant at -40 mV. In the presence of 50 μM Mg^2+^, NMDA peak currents and densities were also significantly larger in transgenic cells (p < 0.01). Similar to the pan-neuronal model, the percent Mg^2+ ^block was smaller in transgenic cells at -70 and -40 mV (p < 0.05, Fig. 5, bottom panel). In the cortical model, NMDA peak currents and densities were similar in WT and transgenic cells and the effects of Mg^2+ ^blockade were not different.

## Discussion

To address the role of pathological cell-cell interactions in striatal pathogenesis in HD mousemodels *in vivo*, we developed a striatum-specific mouse model of HD which expresses a neuropathological fragment of mhtt (mhtt-exon1) selectively in striatal MSNs and in a subset of cortical interneurons but not in the CPNs or other neurons in the brain. This model reveals that striatal-specific expression of mhtt is sufficient to elicit cell-autonomous mhtt nuclear accumulation and aggregation and NMDA receptor dysfunction. However, cell-autonomous toxicity of mhtt is insufficient to elicit progressive locomotor deficits and significant striatal pathology including gliosis and dark neuron degeneration, all of which are observed in the pan-neuronal models of HD [27].

Responses to NMDA and their blockade by Mg^2+ ^were altered in mutant compared to WT mice in the pan-neuronal and striatal model, but not in the cortical model. This indicates that the presence of mhtt in CPNs alone does not lead to changes in NMDA receptor-mediated currents in the striatum. The changes in NMDA receptor function in the pan-neuronal and striatal models were different, with smaller NMDA currents in mutant cells from the pan-neuronal model, and larger NMDA currents in mutant cells from the striatal model. However, in both models Mg^2+ ^blockade of NMDA currents was reduced, similar to effects in R6/2 mice of 15–40 days [31]. Previous studies on dissociated neurons in R6/2 mice have shown both increases and decreases of glutamate receptor-mediated currents depending on the age and the progression of the phenotype. In young R6/2 mice (15–40 days), NMDA and AMPA currents were increased, while in older mice (80 days), they were decreased [31,32]. Similarly, biphasic changes for NMDA and AMPA currents were observed in CPNs in young and older R6/2 mice [33].

At the present time it is difficult to determine the mechanisms underlying this complex set of electrophysiological changes in different models at the same age. The presence of mhtt in striatal neurons appears to be necessary to alter Mg^2+ ^sensitivity. This effect might be related to alterations in NMDA receptor subunits [34-36]. The decrease in magnitude of the NMDA current in the pan-neuronal model might be related to changes in cortical inputs which have been shown to occur in this and other transgenic mouse models of HD [27,37]. In contrast, the increase in current magnitude in the striatal model might reflect the possibility that alterations in the cortex no longer contribute to striatal anomalies.

**Figure 6:**
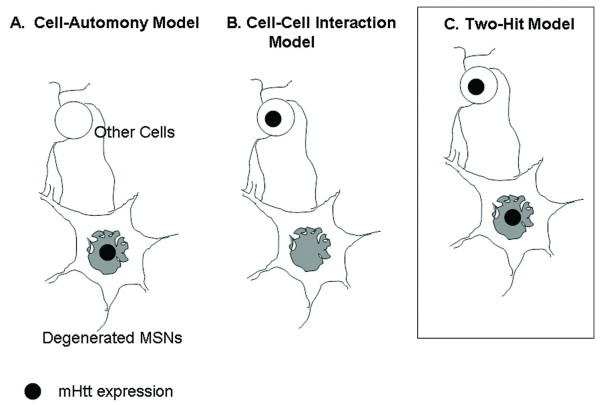
**Schematics illustrating the two-hit hypothesis of neuropathogenesis in conditional models of HD**. Our current analyses of the striatal model and our previous study of the cortical and pan-neuronal model of HD have shown that behavioral deficits and robust cortical and striatal neuropathology are observed only when mhtt-exon1 is broadly expressed in the brain (pan-neuronal model). When mhtt-exon1 only is expressed in one of the known vulnerable neuronal populations, CPNs (cortical model) or striatal MSNs (striatal model), significant motor deficits or robust gliosis and degenerative changes did not occur despite cell-autonomous accumulation of mhtt aggregates. Thus, these findings rule out the cell-autonomous model which suggests expression of mhtt in the vulnerable neurons alone is sufficient to elicit robust neuropathology in that neuron (A). Since in both the cortical model [27] and in the striatal model, cell-autonomous expression of mhtt did exhibit evidence of neuronal dysfunction (*i.e. *degenerating lysosomes in the cortical model and electrophysiological changes in striatal model), the findings also suggest a pure cell-cell interaction model (B) is also unlikely. Thus, our current data favor the two-hit hypothesis (C), which suggests both cell-autonomous toxicity and pathological cell-cell interaction synergistically contribute to neuropathogenesis of the vulnerable cortical and striatal neurons.

The core observations of the present study are consistent with our prior analyses of the cortical model of HD. In both cases, we found that mhtt aggregation is cell-autonomous, but cell-autonomous toxicity of mhtt in either CPNs or MSNs is not sufficient to elicit robust regionally-specific neuropathology and behavioral deficits as compared to the pan-neuronal model. Thus, pathological cell-cell interactions appear to contribute to neuronal toxicity in both vulnerable neuronal populations in HD. Although cell-type specific expression of mhtt-exon 1 is insufficient to elicit robust pathology, our analyses did provide evidence of cell-autonomous neuronal dysfunction. In the cortical model, we showed expression of mhtt-exon1 in CPNs resulted in mild degenerative changes (*i.e. *dark degenerating vacuoles in the CPNs under EM; see ref. [27]); and in the striatal model, electrophysiological recordings revealed cell-autonomous NMDA receptor-mediated response changes in dissociated MSNs. Moreover, the regionally-specific expression of mhtt-exon 1 appears to be insufficient to elicit robust non-cell-autonomous toxicity in other brain regions. For example, selective expression of mhtt-exon1 in CPNs is not sufficient to cause a significant striatal phenotype in the cortical model. Thus, pathological cell-cell interactions are necessary but not sufficient for cortical and striatal pathogenesis in HD. Based on these observations, we propose a ''two-hit'' hypothesis in which mhtt can elicit both cell-autonomous toxicity and pathological cell-cell interactions which are necessary for selective neuronal toxicity in HD (Fig. 6)

Our analyses of the regional models of HD are consistent with prior evidence supporting the possible role of cell-cell interactions in HD striatal pathogenesis. First, excitotoxicity is thought to contribute to striatal pathogenesis in HD. The mechanisms underlying such toxicity are not completely clear, but may include both cell-autonomous mechanisms such as alteration in NMDA receptor function in the MSNs [17,33,38-40] or non-cell-autonomous mechanisms, such as enhanced glutamate release from CPNs [40-43] or reduced glutamate clearance [44-46]. Our *in vitro *electrophysiological analysis clearly confirms prior results with R6/2 mice [23], and suggests that altered Mg^2+ ^blockade of NMDA receptors in striatal MSNs may contribute to striatal excitotoxicity. Another mechanism supporting cell-cell interactions in HD is the role of cortical BDNF in striatal pathogenesis suggesting that the transcription and axonal transport of BDNF is diminished in HD models [25,47]. Since cortex-specific deletion of BDNF is known to elicit progressive striatal degeneration in mice [26], BNDF reduction will contribute to striatal pathogenesis in HD. Finally, non-neuronal cells, particularly astrocytes, are also affected in HD and HD mice. Studies using primary cell culture demonstrate that astrocytes derived from R6/2 mice have reduced capacity to transport glutamate, and thus cannot protect mutant neurons from glutamate toxicity as well as astrocytes of WTs [24]. Studies using mouse models of other neurodegenerative disorders, such as amyotrophic lateral sclerosis [48], frontotemporal dementia [49], synucleinopathy [50], and spinocerebellar ataxia 7 (another polyQ disorder) [51], provide *in vivo *genetic evidence that non-neuronal cells may contribute to neurodegeneration. Thus, converging evidence suggests pathological cell-cell interaction as a common cellular mechanism in multiple neurodegenerative disorders.

Our analyses of the striatal model of HD also indicate that caution should be used when interpreting neurodegenerative phenotypes in HD cellular models over-expressing pathogenic fragments of mhtt. These cellular models have been particularly valuable for rapid dissection of molecular mechanisms underlying the mhtt toxicity. Since neurodegeneration in such models occurs rapidly in days and is due to cell-autonomous toxicity of mhtt, and our striatal model of HD demonstrates that cell-autonomous toxicity of mhtt-exon1 *in vivo *is insufficient to elicit significant neuropathology, the rapid neurodegenerative process observed in cellular models may reflect different mechanisms than the slow degenerative process which occurs in the *in vivo *models. The latter may require pathological cell-cell interactions. We suggest that the cellular models are valuable for rapid identification of candidate molecular mechanisms underlying both cell-autonomous and non-cell-autonomous toxicity of mhtt, and these mechanisms should be validated in *in vivo *genetic models of HD.

## Conclusion

We have developed a novel genetic mouse model in which a neuropathogenic fragment of mhtt-exon1 is selectively expressed in striatal MSNs and a subset of cortical interneurons. We demonstrate that the striatal model of HD exhibits cell-autonomous accumulation of nuclear mhtt aggregates, and alterations in NMDA receptor function, but this model lacks robust striatal neuropathology (*i.e. *gliosis and dark neuron neurodegeneration) and progressive motor deficits. Taken together these findings emphasize that pathological cell-cell interactions are necessary for striatal pathogenesis *in vivo *in genetic mouse models of HD.

## Methods

### Animals

The RosaHD mouse is a conditional HD mouse model in which expression of mhtt-exon1 with 103 glutamine repeats is completely dependent on Cre-mediated excision of a transcription termination sequence flanked by two Cre binding sites (LoxP) (Fig 1A). RosaHD mice were bred with Dlx5/6-Cre mice [29] to generate the striatal model that expresses mhtt in striatal MSNs and cortical interneurons. The pan-neuronal and cortical models were generated by crossing RosaHD mice with Nestin-Cre and Emx-Cre mice as reported previously [27]. RosaHD mice were maintained as 129Sv and C57BL/6 hybrids. Dlx5/6-Cre mice were maintained on C56BL/6 background. The male and female F1 mice from crosses between RosaHD and Cre mice were used in the present experiments. Mice were group housed in a clean facility with free access to food and water. Animal breeding and experiment protocols were in accordance with the National Institute of Health Guidelines for use of live animals and approved by the Animal Research Committee at UCLA.

### Automatic Open Field Test

Twenty naïve transgenic and 22 WT littermates at 6 months of age and 15 transgenic and 14 WT littermates at 12 month were tested with an Automatic Open Field system (Tru Scan 99; Coulbourn Instruments, Allentown, PA). A mouse was placed in an open field arena for 15 min during the dark phase of the 6:00 PM–6:00 AM dark-light cycle. Locomotor activity and the proportion of time spent in the center and margin areas were recorded and calculated using the Tru Scan 99 Software.

### Neuropathology

Transgenic and their WT littermates were transcardially perfused with ice-cold 0.1 M phosphate buffer (PB, pH 7.2), followed by ice cold 4% paraformaldehyde with 0.2% glutaraldehyde in 0.1 M PB (pH 7.4). After 6 h postfix in 4% paraformaldehyde buffer as above, brains were equilibrated with 30% sucrose by shaking at 4°C until the brains sank to the bottom of the container. Thirty-five μm coronal or saggital brain sections were cut using a Leica CM 1850 cryostat and stored at -20°C. Free-floating sections were processed for immunohistochemistry staining as described previously [27]. The following primary antibodies were used: rabbit anti-human htt EM48 antibody (1:300), mouse anti-GFAP antibody (1:10000, Sigma), goat anti-mouse Darpp32 antibody (1:1000, Chemicon), mouse anti-GABA (1: 1000, Sigma). EM48 and GFAP immunostaining were visualized by using diaminobenzidine as the chromogen (Pierce); Goat anti-rabbit Alexa Fluor 488 (Molecular Probes) and goat anti-rabbit Texas-red (DAKO), goat anti-mouse Alexa Flour 546 (Molecular Probes) were used for EM48/Darpp32 and EM48/GABA double immunofluorescent stainings, respectively. The EM48/Darpp32 double immunofluorescence staining was carried out by using the two step sequential method after verification of antibody specificity in control sections. Briefly, brain sections were blocked with normal goat serum and stained with EM48 antibody and Alexa Fluor 488 secondary antibody. The sections were further washed and blocked with normal goat serum, then stained with Darpp32 antibody and Texas-red secondary antibody. All steps in the second round of Darpp32 immunostaining were carried out in the dark. Quantitative analyses of reactive gliosis were performed blind with respect to genotype as described [27].

### Electron Microscopy

Two 12 month-old pan-neuronal, cortical and striatal model mice and their WT littermates were trancardially perfused with ice-cold 0.1 M PB (pH 7.2), followed by ice cold 2% paraformaldehyde with 2% glutaraldehyde in 0.1 M PB (pH 7.4). The brains were dissected out and immersed in 0.1 M PB for 6 h. 100 μm sections were sliced using a vibratome and treated with 1% osmic acid for 2 h. After washing with 0.1 M PB, brain sections were dehydrated in ascending concentrations of ethanol and propylene oxide/Eponate 12(1:1) and embedded in Eponate 12(Ted Pella Inc., Redding, CA). Ultrathin sections (60 nm) were cut using a Leica UltracutS ultramicrotome. Thin sections were counterstained with 5% aqueous uranyl acetate for 5 min followed by Reynolds lead citrate for 5 min and examined using a Hitachi (Tokyo, Japan) H-7500 electron microscope.

### Electrophysiological Recordings

Mice 8–10 months of age were used. The following are the numbers of mice and cells recorded from each group: 4 pan-neuronal model mice (14 cells) and 3 WT littermate controls (10 cells), 4 striatal model mice (21 cells) and 4 WT littermate controls (19 cells), 4 cortical model mice (25 cells) and 4 WT littermate controls (14 cells). Details of procedures for acute dissociation and recording have been published [31]. Briefly, mice were anesthetized with halothane, transcardially perfused and decapitated. Brains were dissected and sliced coronally and the dorsal part of the striatum was dissected for acute dissociation of neurons. Cells were recorded in an external Mg^2+ ^free external solution (in mM): 135 NaCl, 20 CsCl, 5 BaCl_2_, 10 glucose, 10 HEPES, 0.02 glycine and 0.0003 tetrodotoxin (TTX, pH 7.4, 300–310 mOsm). The external recording solution contained Ba^2+^, instead of Ca^2+^, to minimize the influence of Ca^2+^-mediated transduction systems. The presence of Cs^+ ^and TTX in the external solution blocked some voltage-gated K^+ ^and Na^+^channels, respectively. The internal pipette solution contained (in mM): 175 N-methyl-D-glucamine (NMDG), 40 HEPES, 2 MgCl_2_, 10 ethylene glycol-bis (β-aminoethyl ether)-N, N, N', N'-tetraacetic acid (EGTA), 12 phosphocreatine, 2 Na_2_ATP, 0.2 Na_2_GTP and 0.1 leupeptin (pH 7.2–7.3, 265–270 mOsm). Electrode resistance was typically 4–5 MΩ in the bath. After seal rupture, series resistance was compensated (70–90%) and periodically monitored. Only data from cells for which access resistance values were smaller than 20 MΩ were included. Membrane capacitances and input resistances were measured to compare membrane properties between neurons from WT and transgenic mice. Drugs were applied through an array of application capillaries positioned 500–600 μm from the cell using a pressure-driven fast perfusion system. NMDA was applied for 3 sec every 10 sec. Responsiveness of cells to 100 μM NMDA and their sensitivity to 50 μM Mg^2+ ^were examined at two different holding potentials: -70 and -40 mV. The NMDA receptor specific antagonist 2-amino-5-phosphonovalerate was applied to confirm the specificity of NMDA-induced currents (data not shown). To normalize to cell size, peak current densities were measured by dividing peak amplitude values by the capacitance of the cell.

### Statistics

Values in the figures and text are presented as mean ± standard error (SE). Appropriate student's *t*-tests alone were used when two group means were compared. Electrophysiological membrane properties and NMDA current characteristics were compared using appropriate two-way analyses of variance (ANOVAs) with one repeated measure followed by multiple comparisons using Bonferroni *t*-tests. Differences between means were considered statistically significant if P < 0.05.

## Competing interests

The author(s) declare that they have no competing interests.

## Authors' contributions

XG managed the mouse colony, performed the behavioral and neuropathological analyses, and assisted in preparation of the manuscript. VAM and CC carried out the electrophysiological study; SHL and XJL carried out the EM study; MSL directed the electrophysiological studies, and contributed to writing of the manuscript; XWY conceived the project, oversaw the phenotypic analyses of the mutant mice, and contributed to writing of the manuscript. All authors read and approved the final manuscript.
